# Influence of obesity on mortality, mechanical ventilation time and mobility of critical patients with COVID-19

**DOI:** 10.62675/2965-2774.20240253-en

**Published:** 2024-06-19

**Authors:** Luísa Helena Machado Martinato, Débora Schmidt, Taila Cristina Piva, Gracieli Nadalon Deponti, Maricene Colissi Graboski, Rodrigo Della Méa Plentz, Graciele Sbruzzi

**Affiliations:** 1 Hospital de Clínicas de Porto Alegre Universidade Federal do Rio Grande do Sul Porto Alegre RS Brazil Physical Therapy Service, Hospital de Clínicas de Porto Alegre, Universidade Federal do Rio Grande do Sul - Porto Alegre (RS), Brazil.; 2 Postgraduate Program in Rehabilitation Science Universidade Federal de Ciências da Saúde de Porto Alegre Porto Alegre RS Brazil Postgraduate Program in Rehabilitation Science, Universidade Federal de Ciências da Saúde de Porto Alegre - Porto Alegre (RS), Brazil.

**Keywords:** Obesity, COVID-19, SARS-CoV-2, Coronavirus infections, Mortality, Rehabilitation, Respiration, artificial, Intensive care units

## Abstract

**Objective:**

To identify the influence of obesity on mortality, time to weaning from mechanical ventilation and mobility at intensive care unit discharge in patients with COVID-19.

**Methods:**

This retrospective cohort study was carried out between March and August 2020. All adult patients admitted to the intensive care unit in need of ventilatory support and confirmed to have COVID-19 were included. The outcomes included mortality, time on mechanical ventilation, and mobility at intensive care unit discharge.

**Results:**

Four hundred and twenty-nine patients were included, 36.6% of whom were overweight and 43.8% of whom were obese. Compared with normal body mass index patients, overweight and obese patients had lower mortality (p = 0.002) and longer intensive care unit survival (log-rank p < 0.001). Compared with patients with a normal body mass index, overweight patients had a 36% lower risk of death (p = 0.04), while patients with obesity presented a 23% lower risk (p < 0.001). There was no association between obesity and time on mechanical ventilation. The level of mobility at intensive care unit discharge did not differ between groups and showed a moderate inverse correlation with length of stay in the intensive care unit (*r* = -0.461; p < 0.001).

**Conclusion:**

Overweight and obese patients had lower mortality and higher intensive care unit survival rates. The duration of mechanical ventilation and mobility level at intensive care unit discharge did not differ between the groups.

## INTRODUCTION

The coronavirus disease 2019 (COVID-19), caused by severe acute respiratory syndrome coronavirus 2 (SARS-CoV-2), has rapidly evolved into a global pandemic. There are more than 770 million confirmed cases and approximately 6.9 million deaths worldwide.^(1)^ Brazil is the third largest country in terms of the number of confirmed cases (more than 37.7 million) and the second worst in terms of the number of deaths from complications of the disease (exceeding 700 thousand), being identified as the epicenter of the pandemic at several times.^(1)^

The COVID-19 pandemic overlapped with another well-known global epidemic in our society, that of overweight and obesity. Several meta-analyses have examined the relationship between obesity and adverse outcomes and have revealed an increased risk for hospitalization,^(2-4)^intensive care unit (ICU) admission,^(2-6)^the need for invasive mechanical ventilation (MV),^(2-4,6)^and hospital death.^(2-5)^However, heterogeneity measures of the death outcome were moderate or substantial, and some authors argue in favor of the existence of an “obesity paradox,” in which overweight individuals have an increased risk of developing the severe form of the disease while mortality is similar or lower than that of patients without obesity.^(7-10)^

Studies on COVID-19 have focused on improving the survival of patients in intensive care, with insufficient research on morbidities and functional limitations related to this disease in its critical form resulting from prolonged immobilization in bed, long stays on MV and all ICU support. In different profiles of critically ill patients, mobilization is safe, feasible and beneficial.^(11)^ In non-COVID-19 ICUs, there are potential barriers that make rehabilitation difficult,^(12)^ and the pandemic has brought additional challenges, such as the need for strict infection control measures,^(13)^ the limitation of physical and human resources to assist in rehabilitation, the high proportion of patients with a high body mass index (BMI),^(14)^ and the constant need to free up beds to meet an increasing demand for new admissions. Thus, the research focus is placed on stability, survival and early discharge from the ICU and hospital, with rehabilitation, especially in the ICU, potentially being neglected.^(14)^

To date, the published data regarding hospital mortality of the obese population admitted to the ICU for COVID-19 are controversial, and there is insufficient evidence regarding the time this population remains on MV and their mobility when they are discharged from the ICU. The fact that obesity is highly prevalent among the population and is a risk factor for the need for hospitalization and MV is a concern. Thus, the objective of this study was to identify the influence of obesity on mortality, time on MV and level of mobility at ICU discharge in patients with COVID-19.

## METHODS

### Study design

This retrospective cohort study utilized the medical records of patients with confirmed SARS-CoV-2 infection admitted consecutively from March 1 to August 31, 2020, at the COVID-19 ICUs of *Hospital de Clínicas de Porto Alegre*, a reference for the care of highly complex COVID-19 patients in Rio Grande do Sul, Brazil. The Institutional Research Ethics Committee approved this study (CAAE: 35513220.5.0000.5327), which follows the Declaration of Helsinki. The observational and retrospective nature of the research waived the need for an informed consent form.

### Subjects

The sample included patients with a diagnosis of COVID-19 confirmed by reverse transcription polymerase chain reaction (RT‒PCR), of both sexes, aged older than 18 years, admitted to the COVID-19 ICU and in need of ventilatory support (invasive or noninvasive) for more than 24 hours. Patients with incomplete data in their medical records that made it impossible to assess their BMI, with previous functional limitations or who were transferred to another hospital while on MV were excluded from the study. This study was conducted during the first wave of the COVID-19 pandemic when vaccines were still under development, so the patients included were not vaccinated.

### Data collection

Data collection was carried out with a thorough reading and review of the patient’s electronic medical records by the researchers, which were extracted to a spreadsheet with access restricted to the study collaborators. Demographic and anthropometric data, previous comorbidities, smoking status and alcohol consumption, and severity scores were collected using the Simplified Acute Physiology Score 3 (SAPS 3). Regarding therapies, data on ventilatory support, hemodialysis, extracorporeal membrane oxygenation (ECMO), nitric oxide (NO), prone position, sedatives, neuromuscular blockers, and corticosteroids were collected. To characterize the clinical evolution in the ICU, we verified the length of stay in the ICU and the hospital, the duration of bed rest (defined as the days elapsed between admission to the ICU and the first time out of bed), mobility scores, MV time, length of hospital stay and ICU mortality.

Obesity was defined according to BMI, with weight and height measured in the first 24 hours of ICU admission by the nutritionist in charge. Patients were classified into three groups according to the classification proposed by the World Health Organization (WHO): normal BMI (BMI 18.5 - 24.9kg/m^2^), overweight (BMI 25 - 29.9kg/m^2^), and obese (BMI ≥ 30kg/m^2^).^(15)^

### Outcomes

The primary outcome was ICU mortality. Secondary outcomes included time on MV, defined as the days elapsed between the start and end of invasive ventilatory support, and the level of mobility assessed at the last ICU physical therapy visit, using the following scores:

- The Perme Intensive Care Unit Mobility Score comprises 15 items grouped into seven categories (mental status, potential mobility barriers, functional strength, mobility in bed, transfers, gait, and resistance). The final score ranges from 0 to 32, with a high score indicating few potential mobility barriers and less need for assistance.^(16)^
- The Intensive Care Unit Mobility Scale is a single domain instrument scored from 0 to 10, with a score of 0 being interpreted as a patient able to perform only passive exercises in bed and a score of 10 meant the patient can walk unaided.^(16)^

### Statistical analysis

The normality of the data distribution was assessed by visual inspection of histograms and Q-Q plots. Categorical variables are described as frequencies and percentages, while continuous variables are presented as the mean and standard deviation or median and quartiles 1 and 3. Pearson’s chi-square test of independence and Fischer’s exact test were used to compare the categorical variables for demographic data and clinical characteristics between the three BMI groups. To verify the associations of continuous variables between groups, we used one-way ANOVA for normally distributed data and the Kruskal–Wallis test for nonparametric data. When necessary, a *post hoc* analysis of multiple comparisons with Bonferroni correction was performed. Survival times between BMI groups were compared using Kaplan‒Meier estimates and the log-rank test for the equality of survival curves. Correlations between variables were established using Spearman’s correlation coefficient. To analyze the associations between BMI and ICU mortality, a Poisson regression model with robust variance was used for univariate and multivariate analyses. The results are given as the relative risk and 95% confidence interval (95%CI). The software used was the Statistical Package for Social Sciences (SPSS), version 18, and the significance level adopted was 5%.

## RESULTS

**Figure 1:**
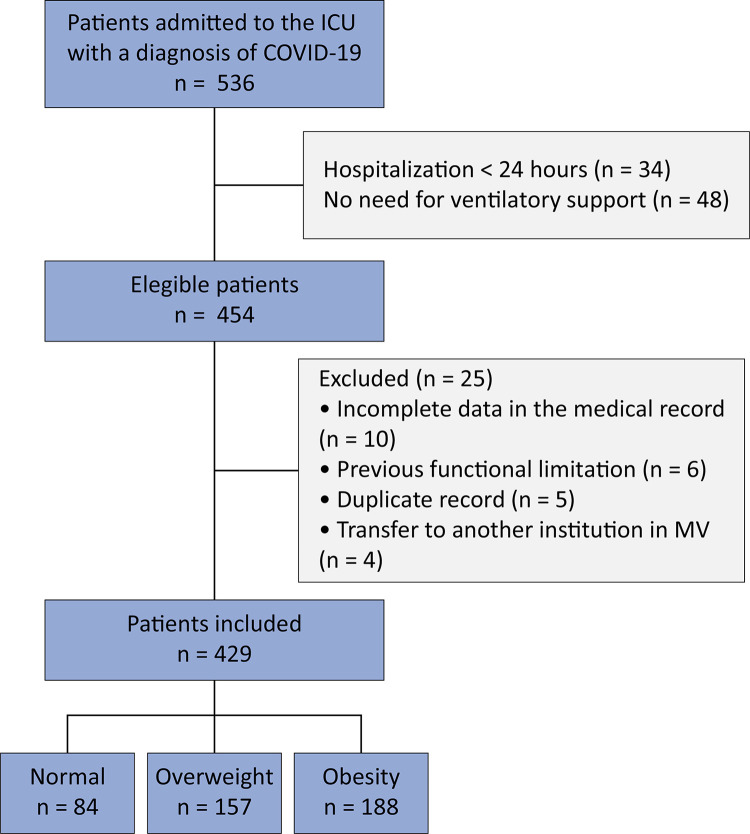
Selection of patients included in the study.

During the study period, 536 patients were admitted to the ICU with a diagnosis of COVID-19 and 454 were eligible. Of these, 429 patients were included: 43.8% were obese, 36.6% were overweight, and 19.6% had a normal BMI (Figure 1).

ICU - intensive care unit; MV - mechanical ventilation.

The average overall age was 58 years; overweight and obese patients were younger and presented a lower severity score upon ICU admission through the SAPS 3 than patients with a normal BMI. The sample characterization data, instituted therapies, mobility, and mortality data are presented in table 1.

**Table 1:** Characteristics of the study population

	n	Total n = 429	Normal BMI n = 84	Overweight n = 157	Obesity n = 188	p value
Age (years)	429	58.4 ± 13.8	65.2 ± 12.8	57.7 ± 13.7	56.0 ± 13.4	< 0.001
Male	429	250 (58.3)	54 (64.3)	106 (67.5)	90 (47.9)	0.001
White	429	338 (79.0)	69 (82.1)	123 (78.3)	146 (78.1)	0.87
SAPS 3	429	55.7 ± 12.8	60 ± 13.0	56 ± 13.3	54 ± 12.0	0.001
Comorbidities						
Hypertension	429	262 (61.1)	45 (53.6)	89 (56.7)	128 (68.1)	0.03
Diabetes mellitus	429	168 (39.2)	30 (35.7)	64 (40.8)	74 (39.4)	0.74
Heart disease	429	87 (20.3)	28 (33.3)	31 (19.7)	28 (14.9)	0.002
Chronic kidney failure	429	65 (15.2)	20 (23.8)	22 (14.0)	23 (12.2)	0.04
Smoking	429	111 (25.9)	29 (34.5)	40 (25.5)	42 (22.3)	0.11
Alcoholism	429	26 (6.1)	9 (10.7)	11 (7.0)	6 (3.2)	0.046
Therapies						
Corticosteroid	429	399 (93.0)	78 (92.9)	143 (91.1)	178 (94.7)	0.426
Sedation	429	371 (86.5)	76 (90.5)	140 (89.2)	155 (82.4)	0.094
Neuromuscular blocker	358	294 (82.1)	54 (73.0)	109 (80.1)	131 (88.5)	0.01
Prone position	358	171 (47.8)	23 (31.1)	65 (47.8)	83 (56.1)	0.002
Hemodialysis	429	164 (38.2)	35 (41.7)	61 (38.9)	68 (36.2)	0.68
Nitric oxide	358	20 (5.6)	0 (0.0)	11 (8.1)	9 (6.1)	0.048
ECMO	358	11 (3.1)	0 (0.0)	6 (4.4)	5 (3.4)	0.19
Ventilatory support						
HFNC	429	92 (21.5)	14 (16.7)	34 (21.8)	44 (23.4)	0.46
NIV	429	154 (35.9)	34 (40.5)	48 (30.6)	72 (38.3)	0.20
MV	429	358 (83.4)	74 (88.1)	136 (86.6)	148 (78.7)	0.06
Overall time on MV	355	15 (8 - 26)	13 (7 - 22)	15 (8 - 29)	16 (10 - 28)	0.08
Time on MV (survivors)	175	14 (7 - 27)	12 (7 - 24)	13 (6 - 30)	15 (9 - 26)	0.45
Mobility						
ICU Mobility Scale	248	5 (4 - 7)	5 (4 - 7)	5 (4 - 7)	5 (4 - 8)	0.63
Perme Score	236	20 ± 8	19 ± 8	20 ± 8	20 ± 8	0.73
Bed rest days	250	8 (3 - 18)	6 (2 - 16)	8 (3 - 19)	9 (3 - 20)	0.40
ICU mortality	429	182 (42.4)	52 (61.9)	57 (36.3)	73 (38.3)	0.002
Length of ICU stay	429	15 (8 - 26)	13 (8 - 25)	15 (8 - 28)	14 (8 - 26)	0.43
Hospital mortality	429	195 (45.5)	57 (67.9)	61 (38.9)	77 (39.5)	< 0.001
Length of hospital stay	429	22 (13 - 36)	21 (13 - 34)	23 (14 - 39)	22 (13 - 35)	0.45

BMI - body mass index; SAPS 3 - Simplified Acute Physiology Score III; ECMO - extracorporeal membrane oxygenation; HFNC - high-flow nasal cannula; NIV - noninvasive mechanical ventilation; MV - mechanical ventilation. The results are presented as the n (%), mean ± standard deviation and median (Q1 - Q3).

**Figure 2:**
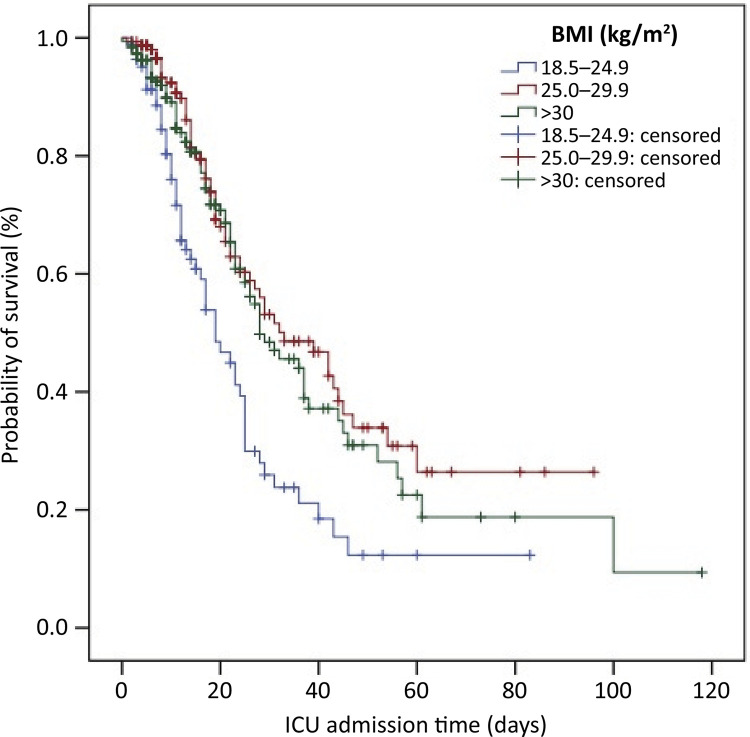
Kaplan–Meier curve for intensive care unit survival time by body mass index group (log-rank p < 0.001).

The mortality rate in the ICU reached 42.4%, and in patients with a normal BMI, it was 61.9%. Compared with patients with a normal BMI, overweight and obese patients had longer ICU survival times (log-rank < 0.001) (Figure 2). According to the Poisson multivariable regression model, overweight and obesity were significant protective factors against mortality in the ICUs of patients who required MV (p = 0.04 and p < 0.001, respectively). Thus, overweight patients had a 36% lower risk of progressing to death than did patients with a normal BMI, while in patients with obesity, the rate was 23%. The SAPS 3 score was presented as an independent risk factor for mortality (p < 0.001), and each additional point in the score represented a 2% risk of death in the ICU for patients who required MV. In the univariate analysis, age and diabetes were risk factors for mortality (p < 0.001 and p = 0.02, respectively), but they lost effect after adjustments of the multivariate analysis. The time on MV did not contribute to ICU mortality (Table 2).

BMI - body mass index; ICU - intensive care unit.

**Table 2:** Adjusted relative risks for the association between body mass index category and mortality only for patients on mechanical ventilation

Variable	n	Mortality	Innivariable		Multivariable	
			Relative risk (95%CI)	p value	Relative risk (95%CI)	p value
Age	346	181	1.02 (1.01 - 1.02)	< 0.001	1.01 (1.00 - 1.02)	0.18
Women	346	181	0.97 (0.79 - 1.19)	0.78	0.93 (0.76 - 1.13)	0.45
Diabetes	346	181	1.26 (1.03 - 1.54)	0.02	1.19 (0.95 - 1.48)	0.13
Hypertension	346	181	1.17 (0.94 - 1.45)	0.16	0.97 (0.76 -1.24)	0.80
SAPS 3	346	181	1.02 (1.01 - 1.03)	< 0.001	1.02 (1.01 - 1.03)	< 0.001
Time on MV*	346	181	1.06 (0.94 - 1.20)	0.37	1.4 (1.00 - 1.29)	0.054
BMI	346	181				
18.5 - 24.99kg/m^2^			1.0 (reference)		1.0 (reference)	
25 - 29.99kg/m^^2^^			0.59 (0.46 - 0.78)	< 0.001	0.64 (0.5 0- 0.82)	< 0.001
≥ 30kg/m^^2^^			0.63 (0.49 - 0.80)	< 0.001	0.77 (0.61 - 0.97)	0.02

95%CI - 95% confidence interval; SAPS 3 - Simplified Acute Physiology Score 3; MV - mechanical ventilation; BMI - body mass index.

* Mechanical ventilation days in log.

**Figure 3:**
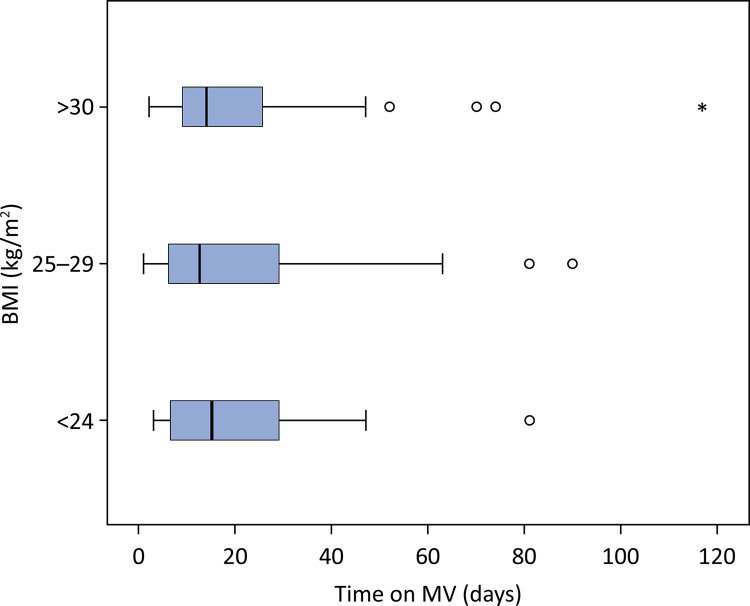
Time on mechanical ventilation in survivors according to body mass index group.

Overall, 83.4% of patients required MV for an overall median time on MV of 15 days (8 - 26), and there was no difference among the three groups (Figure 3). In survivors, the time on MV did not differ between groups (Table 1).

BMI - body mass index; MV - mechanical ventilation.

**Figure 4:**
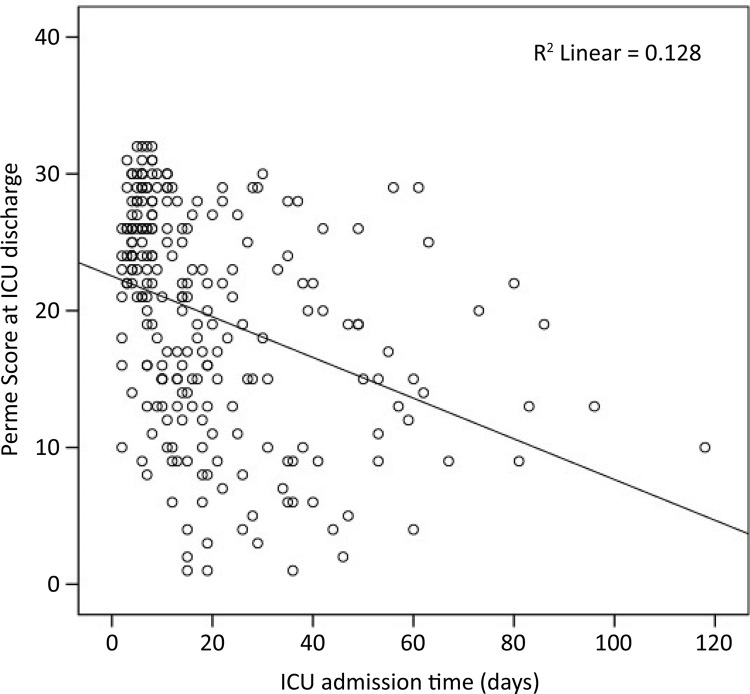
Correlation of the Perme score at intensive care unit discharge with length of stay in the intensive care unit (r = -0.461, p < 0.001).

When comparing mobility data, no differences were observed among patients who were obese, overweight or had a normal BMI (Table 1). The median time for the first bed exit in all groups was eight days (3 - 18). At the time of ICU discharge, the average Perme score was 20 ± 8 points, indicating that a higher score corresponds to a lower need for assistance in mobilization. The achieved level of mobility on the ICU Mobility Scale was 5 (4 - 7), meaning that 50% of patients were capable of actively transferring weight from one leg to another up to a chair. There was no association between BMI and mobility at ICU discharge (Perme Score and ICU Mobility Scale) or between BMI and the duration of bed exit. However, the length of ICU stay showed a moderate inverse correlation with the Perme score (r = -0.461, p < 0.001) (Figure 4), and the pattern of this association remained similar when the groups were analyzed separately.

ICU - intensive care unit.

## DISCUSSION

This single-center study evaluated the influence of obesity on mortality, time on MV, and level of mobility at ICU discharge in critically ill patients diagnosed with COVID-19. In addition to having lower mortality rates, overweight and obese patients also had longer ICU survival times than did patients with a normal BMI. Overweight and obesity were found to be protective factors for predicting ICU mortality after adjustment for confounders. There was no association between obesity and time on MV. The level of mobility at ICU discharge did not differ between the groups and showed a moderate inverse correlation with the length of stay in the ICU.

The prevalence of obese patients admitted to the ICU for COVID-19 was high in the present study (43.8%), as also described in previous publications.^(17-20)^Obesity is an independent factor associated with an increased risk of ICU admission for COVID-19.^(20)^ In a retrospective cohort, approximately half of the individuals admitted to the ICU with SARS-CoV-2 had a BMI ≥ 30kg/m^2^, which is different from the distribution observed in previous years of hospitalization for pulmonary causes, in which the prevalence of obesity was only 25.8%.^(18)^ In the study by van der Voort (2020), 90% of patients infected with SARS-CoV-2 with respiratory failure had a BMI greater than 25kg/m^2^ and the severity of the disease increased significantly with a high BMI.^(21)^ In a pair-matched 1:2 case-control study, patients with obesity were more likely to develop severe SARS‒CoV‒2 disease and require intensive treatment after confounding factors were excluded.^(22)^

Obesity can increase the severity of the disease and potentially predispose younger individuals to the need for hospitalization and ICU admission.^(18,23)^Although we did not assess the risk of ICU admission, in addition to the high prevalence of obesity, overweight patients were younger than patients with a normal BMI. Kass et al. reported an inverse correlation between age and BMI in a population of 265 patients admitted to the ICU with COVID-19, in which younger individuals admitted to the hospital were more likely to be obese.^(24)^

The association between obesity and a greater risk of death is controversial. Some studies argue for the existence of an “obesity paradox,” in which patients with obesity have an increased risk of critical illness but similar mortality compared to patients without obesity.^(7-10,20)^Before our study, some retrospective cohorts analyzed the mortality of obese patients in the ICU and, similar to our data, found that the percentage of survivors was greater^(9)^ or similar^(10,25-27)^for obese patients. This lower mortality rate in obese patients in the ICU may be related to the obesity paradox as a protective effect or to the selection bias of less severely obese patients admitted to the ICU and limitations inherent to observational research. The ICUs of the present study are part of the reference hospital for high-complexity care for patients with COVID-19 in Rio Grande do Sul, Brazil. Thus, patients with greater severity and/or patients believed to have greater potential for complications were referred for highly complex treatment, as were patients with greater potential for recovery.

Several meta-analyses have examined the relationship between obesity and an increased risk of in-hospital death,^(2-6,28)^however, measures of death outcome heterogeneity are moderate or substantial. The most recent systematic review that addressed the effect of obesity on mortality revealed high-certainty evidence that class III obesity is associated with an increased risk of death in patients with COVID-19, but in mild cases of obesity (classes I and II), this factor may not be independently associated.^(28)^

Regarding the time on MV, we observed, as in other studies, the need for prolonged time in patients with COVID-19.^(29)^ However, this demand for longer MV time was not associated with obesity, as in the study by Pouwels et al., which even reported duration of MV similar to our data: 14 (8 - 23) days.^(25)^ Kooistra et al., despite finding no difference in time on MV between patients with and without obesity, reported a median MV duration of 22 (16 - 40) days.^(26)^

Data on mobility immediately after critical illness in patients with COVID-19 are limited, probably reflecting the nature of the pandemic, when the priority is stabilization and survival of these patients. The study by Medrinal et al. revealed that the mobility levels at ICU discharge were similar to ours and concluded, using the Medical Research Council score, that 69% of COVID-19 ICU survivors developed acquired muscle weakness in the ICU.^(30)^ A survey conducted at the same hospital as the present study revealed that critically ill patients with COVID-19 who developed acquired muscle weakness in the ICU showed compromised strength and mobility at hospital discharge and increased functional dependency six months after ICU discharge.^(31,32)^ A retrospective study in Italy highlighted significant functional impairment in this patient profile, with substantial improvement at hospital discharge through an early rehabilitation program.^(33)^

Obese patients represent an additional challenge for rehabilitation within the ICU. The prospective study by McWillians et al. described the rehabilitation of 110 COVID-19 survivors who required MV and observed a significant impact of BMI on time before first mobilization: patients with a BMI > 40kg/m^2^ took an average of 8 days longer to sit at the bedside for the first time, compared with those with a BMI < 25kg/m^2^.^(14)^This delay was not observed in our study; there was no difference in the median number of days of bed rest between different BMI groups, even with the severity evidenced by the higher prevalence of neuromuscular blocker use and prone position in patients with a BMI > 25kg/m^2^. It should be noted that rehabilitation started early, with patients still intubated, as the median bed rest time was eight days and the median duration of mechanical ventilation among survivors was 14 days.

At the time of ICU discharge, 50% of our patients reached the level of mobility to transfer from the bed to the chair, similar to the findings of studies by McWilliams et al. and Medrinal et al.^(14,30)^Mobility levels with high dependence at ICU discharge even at the start of early rehabilitation may reflect the severity of the disease with the need for prolonged MV, sedatives, and neuromuscular blockers. Another factor to be considered is that the MV and ICU length of stay is very close, with medians of 14 and 15 days, respectively, suggesting that ICU discharge occurs early post-extubation to release critical beds due to the unprecedented burden on the health system. Mobility in our study was inversely associated with the length of ICU stay. In a study by Timenetsky et al., patients in the group that showed improved mobility at ICU discharge had shorter ICU stays and a shorter duration of mechanical ventilation.^(34)^

Our study has limitations. First, there is the potential for residual confounding due to the observational design. Mortality can be influenced by many other factors potentiated by the pandemic, such as socioeconomic disparities, advance directives and health decisions, difficulty accessing the health system and overcrowding. Second, we did not measure the severity of pulmonary involvement using the oxygenation index or other tests. Third, data on mobility levels were collected only at ICU discharge, limiting any conclusions about overall physical recovery. In addition, the study was carried out at a single hospital and may not be representative of other populations. Fourth, the study was conducted during the first wave of the pandemic, when SARS-CoV-2 vaccines were under development. Although this was a single-center study, the institution was the reference center for intensive care of patients with COVID-19.

Despite these limitations, this study also has notable strengths: it has a considerable sample size; it uses relative risks as opposed to odds ratios, which are often calculated in other studies and may exaggerate the risk ratio; it is one of the few to assess the relationship between obesity and mobility in patients with COVID-19; and the main outcome considerations refer exclusively to patients who have completed their stay in the ICU, which allows conviction in the conclusions. Future research should systematically assess strength and functionality for better conclusions about overall physical recovery, further aiding health decisions. Additionally, studies should better assess the impact of overweight/obesity, particularly on health outcomes such as mortality, as most of the evidence encompasses this category in individuals with a normal BMI.

## CONCLUSION

The results suggest that overweight and obese patients have lower mortality and longer survival times in the intensive care unit than patients with a normal body mass index, although they are more often in need of “aggressive” therapies during SARS-CoV-2 infection. Patients with COVID-19 who required mechanical ventilation for a prolonged period, regardless of the high body mass index range, were inversely associated with the length of intensive care unit stay, but overweight and obesity did not influence the level of mobility or assistance. In conclusion, it is recommended that obese patients be monitored to prevent unfavorable clinical and functional outcomes through the implementation of supportive therapies and early initiation of rehabilitation programs.
