# Supplementary material for: Influence of obesity on mortality, mechanical ventilation time and mobility of critical patients with COVID-19
Source: Crit Care Sci. 2024 Jun 19;36:e20240253en. doi: 10.62675/2965-2774.20240253-en (PMC11208042; doi:10.62675/2965-2774.20240253-en)
Supplement: Translation

# Translation: Influência da obesidade na mortalidade, na duração da ventilação mecânica e na mobilidade de pacientes críticos com COVID-19

**DOI:** 10.62675/2965-2774.20240253-pt

**Authors:** Luísa Helena Machado Martinato, Débora Schmidt, Taila Cristina Piva, Gracieli Nadalon Deponti, Maricene Colissi Graboski, Rodrigo Della Méa Plentz, Graciele Sbruzzi

**Affiliations:** 1 Brasil Serviço de Fisioterapia, Hospital de Clínicas de Porto Alegre, Universidade Federal do Rio Grande do Sul - Porto Alegre (RS), Brasil.; 2 Brasil Programa de Pós-Graduação em Ciências da Reabilitação, Universidade Federal de Ciências da Saúde de Porto Alegre - Porto Alegre (RS), Brasil.

**Keywords:** Obesidade, COVID-19, SARS-CoV-2, Infecções por coronavírus, Mortalidade, Reabilitação, Respiração artificial, Unidades de terapia intensiva

## Abstract

**Objetivo:**

Identificar a influência da obesidade na mortalidade, no tempo de desmame da ventilação mecânica e na mobilidade na alta da unidade de terapia intensiva em pacientes com COVID-19.

**Métodos:**

Trata-se de estudo de coorte retrospectivo realizado entre março e agosto de 2020. Foram incluídos todos os pacientes adultos internados na unidade de terapia intensiva com necessidade de suporte ventilatório e diagnosticados com COVID-19. Os desfechos incluíram mortalidade, duração da ventilação mecânica e mobilidade na alta da unidade de terapia intensiva.

**Resultados:**

Identificamos 429 pacientes, dos quais 36,6% estavam acima do peso e 43,8% eram obesos. Em comparação com os pacientes com índice de massa corporal normal, os pacientes com sobrepeso e obesidade apresentaram menor mortalidade (p = 0,002) e maior sobrevida na unidade de terapia intensiva (*log-rank* p < 0,001). Em comparação com pacientes com índice de massa corporal normal, aqueles com sobrepeso tiveram risco 36% menor de morte (p = 0,04), enquanto os pacientes com obesidade apresentaram risco 23% menor (p < 0,001). Não houve associação entre obesidade e duração da ventilação mecânica. O nível de mobilidade na alta da unidade de terapia intensiva não diferiu entre os grupos e apresentou correlação inversa moderada com o tempo de internação na unidade de terapia intensiva (r = -0,461; p < 0,001).

**Conclusão:**

Os pacientes com sobrepeso e obesidade tiveram menor mortalidade e maior tempo de sobrevida na unidade de terapia intensiva. A duração da ventilação mecânica e o nível de mobilidade na alta da unidade de terapia intensiva não diferiram entre os grupos.

## INTRODUÇÃO

A doença pelo coronavírus 2019 (COVID-19), causada pelo coronavírus da síndrome respiratória aguda grave 2 (SARS-CoV-2), evoluiu rapidamente para uma pandemia global. São mais de 770 milhões de casos confirmados e aproximadamente 6,9 milhões de mortes em todo o mundo.^(1)^ O Brasil é o terceiro maior país em número de casos confirmados (mais de 37,7 milhões) e o segundo pior em número de mortes por complicações da doença (mais de 700 mil), sendo identificado como o epicentro da pandemia em diversos momentos.^(1)^

A pandemia da COVID-19 coincidiu com outra epidemia global bem conhecida em nossa sociedade, a do sobrepeso e da obesidade. Várias metanálises examinaram a relação entre obesidade e desfechos adversos e revelaram risco maior de hospitalização,^(2-4)^ internação em unidade de terapia intensiva (UTI),^(2-6)^ necessidade de ventilação mecânica (VM) invasiva^(2-4,6)^ e morte hospitalar.^(2-5)^ No entanto, as medidas de heterogeneidade do desfecho de morte foram moderadas ou substanciais, e alguns autores argumentam a favor da existência de um “paradoxo da obesidade”, no qual indivíduos com sobrepeso apresentam um risco maior de desenvolver a forma grave da doença, enquanto a mortalidade é semelhante ou menor do que a de pacientes não obesos.^(7-10)^

Os estudos sobre a COVID-19 têm se concentrado na melhora da sobrevida dos pacientes em terapia intensiva, sendo insuficientes as pesquisas sobre as morbidades e as limitações funcionais relacionadas a essa doença em sua forma crítica decorrentes da imobilização prolongada no leito, das longas permanências em VM e de todo suporte na UTI. Em diferentes perfis de pacientes críticos, a mobilização é segura, viável e benéfica.^(11)^Nas UTIs não COVID-19, existem potenciais barreiras que dificultam a reabilitação,^(12)^ e a pandemia trouxe desafios adicionais, como a necessidade de medidas rigorosas de controle de infecções,^(13)^ a limitação de recursos físicos e humanos para auxiliar na reabilitação, a elevada proporção de pacientes com um alto índice de massa corporal (IMC)^(14)^ e a necessidade constante de liberar leitos para atender à crescente demanda por novas internações. Assim, o foco principal é colocado na estabilidade, na sobrevivência e na alta precoce da UTI e do hospital, sendo a reabilitação, especialmente na UTI, potencialmente negligenciada.^(14)^

Até o momento, são controversos os dados publicados sobre a mortalidade hospitalar da população obesa internada na UTI por COVID-19 e não há evidências suficientes sobre o tempo que essa população permanece em VM ou sua mobilidade na alta da UTI. O fato de a obesidade ser altamente prevalente na população e ser fator de risco para a necessidade de hospitalização e VM é preocupante. Assim, o objetivo deste estudo foi identificar a influência da obesidade na mortalidade, no tempo de VM e no nível de mobilidade na alta da UTI em pacientes com COVID-19.

## MÉTODOS

### Desenho do estudo

Este estudo de coorte retrospectivo utilizou prontuários de pacientes com infecção confirmada por SARS-CoV-2 internados consecutivamente de 1º de março a 31 de agosto de 2020 nas UTIs COVID-19 do Hospital de Clínicas de Porto Alegre, referência no atendimento de pacientes com COVID-19 de alta complexidade no Rio Grande do Sul. O Comitê de Ética em Pesquisa Institucional aprovou este estudo (CAAE: 35513220.5.0000.5327), que segue a Declaração de Helsinque. A natureza observacional e retrospectiva da pesquisa dispensou a necessidade do Termo de Consentimento Livre e Esclarecido.

### Sujeitos

A amostra incluiu pacientes com diagnóstico da COVID-19 confirmado por transcrição reversa seguida de reação em cadeia da polimerase (RT-PCR), de ambos os sexos, com idade superior a 18 anos, internados na UTI COVID-19 e com necessidade de suporte ventilatório (invasivo ou não invasivo) por mais de 24 horas. Foram excluídos do estudo pacientes com dados incompletos em prontuários que impossibilitassem a avaliação do IMC, com limitações funcionais prévias ou que foram transferidos para outro hospital durante a VM. Este estudo foi realizado durante a primeira onda da pandemia da COVID-19, quando as vacinas ainda estavam em desenvolvimento, de modo que os pacientes incluídos não tinham sido vacinados.

### Coleta de dados

Os pesquisadores realizaram a coleta de dados analisando e lendo minuciosamente os prontuários médicos eletrônicos dos pacientes, que foram extraídos para uma planilha com acesso restrito aos colaboradores do estudo. Foram coletados dados demográficos e antropométricos, comorbidades prévias, tabagismo e consumo de álcool e escore de gravidade através do *Simplified Acute Physiology Score 3* (SAPS 3). Com relação às terapias, foram coletados dados sobre suporte ventilatório, hemodiálise, oxigenação por membrana extracorpórea (ECMO), óxido nítrico, posicionamento em decúbito ventral, sedativos, bloqueadores neuromusculares e corticosteroides. Para caracterizar a evolução clínica na UTI, verificamos o tempo de internação na UTI e no hospital, o tempo de repouso no leito (definido como os dias decorridos entre a admissão à UTI e a primeira saída do leito), os escores de mobilidade, a duração da VM, o tempo de internação hospitalar e a mortalidade na UTI.

A obesidade foi definida de acordo com o IMC, com peso e altura medidos nas primeiras 24 horas de admissão à UTI pelo nutricionista responsável. Os pacientes foram classificados em três grupos, de acordo com a classificação proposta pela Organização Mundial da Saúde (OMS): IMC normal (IMC 18,5 - 24,9kg/m^2^), sobrepeso (IMC 25 - 29,9kg/m^2^) e obesidade (IMC ≥ 30kg/m^2^).^(15)^

### Desfechos

O desfecho primário foi a mortalidade na UTI. Os desfechos secundários incluíram a duração da VM, definida como os dias decorridos entre o início e o fim da VM invasiva e o nível de mobilidade avaliado no último atendimento fisioterapêutico na UTI, usando os seguintes escores:

- O Perme Escore compreende 15 itens agrupados em sete categorias (estado mental, possíveis barreiras à mobilidade, força funcional, mobilidade no leito, transferências, marcha e resistência). A pontuação final varia de zero a 32, sendo que uma pontuação alta indica poucas barreiras potenciais de mobilidade e menor necessidade de assistência.^(16)^
- A Escala de Mobilidade em UTI é um instrumento de domínio único com pontuação de zero a dez, sendo que uma pontuação de zero é interpretada como um paciente capaz de realizar apenas exercícios passivos no leito e uma pontuação de dez significa que o paciente pode caminhar sem ajuda.^(16)^

### Análise estatística

A normalidade da distribuição dos dados foi avaliada por inspeção visual de histogramas e gráficos Q-Q. As variáveis categóricas são descritas como frequências e porcentagens, enquanto as contínuas são apresentadas como média e desvio-padrão ou mediana e quartis 1 e 3. O teste de independência do qui-quadrado de Pearson e o teste exato de Fischer foram usados para comparar as variáveis categóricas dos dados demográficos e das características clínicas entre os três grupos de IMC. Para verificar as associações de variáveis contínuas entre os grupos, usamos a análise de variância (Anova) unidirecional para dados normalmente distribuídos e o teste de Kruskal-Wallis para dados não paramétricos. Quando necessário, realizou-se análise *post hoc* de comparações múltiplas com correção de Bonferroni. Os tempos de sobrevida entre os grupos de IMC foram comparados usando estimativas de Kaplan-Meier e o teste *log-rank* para a igualdade das curvas de sobrevida. As correlações entre as variáveis foram estabelecidas usando o coeficiente de correlação de Spearman. Na análise das associações entre IMC e mortalidade na UTI, foi usado um modelo de regressão de Poisson com variância robusta para análises univariadas e multivariadas. Os resultados são apresentados como risco relativo e intervalo de confiança de 95% (IC95%). Utilizou-se o *Statistical Package for Social Sciences* (SPSS), versão 18, e adotou-se o nível de significância de 5%.

## RESULTADOS

Durante o período do estudo, 536 pacientes foram admitidos na UTI com o diagnóstico da COVID-19, sendo 454 elegíveis. Desses, foram incluídos 429 pacientes: 43,8%com obesidade, 36,6% com sobrepeso e 19,6% tinham IMC normal (Figura 1).

**Figura 1:** Seleção dos pacientes incluídos no estudo. UTI - unidade de terapia intensiva; VM - ventilação mecânica.

A média de idade geral foi de 58 anos; os pacientes com sobrepeso e obesidade eram mais jovens e apresentaram escore de gravidade mais baixo na admissão à UTI por meio do SAPS 3 do que os pacientes com IMC normal. Os dados de caracterização da amostra, as terapias instituídas, a mobilidade e os dados de mortalidade são apresentados na tabela 1.

**Tabela 1:** Características da população do estudo

	n	Total n = 429	IMC normal n = 84	Sobrepeso n = 157	Obesidade n = 188	Valor de p
Idade (anos)	429	58,4 ± 13,8	65,2 ± 12,8	57,7 ± 13,7	56,0 ± 13,4	< 0,001
Masculino	429	250 (58,3)	54 (64,3)	106 (67,5)	90 (47,9)	0,001
Branca	429	338 (79,0)	69 (82,1)	123 (78,3)	146 (78,1)	0,87
SAPS 3	429	55,7 ± 12,8	60 ± 13,0	56 ± 13,3	54 ± 12,0	0,001
Comorbidades						
Hipertensão	429	262 (61,1)	45 (53,6)	89 (56,7)	128 (68,1)	0,03
*Diabetes mellitus*	429	168 (39,2)	30 (35,7)	64 (40,8)	74 (39,4)	0,74
Cardiopatia	429	87 (20,3)	28 (33,3)	31 (19,7)	28 (14,9)	0,002
Insuficiência renal crônica	429	65 (15,2)	20 (23,8)	22 (14,0)	23 (12,2)	0,04
Tabagismo	429	111 (25,9)	29 (34,5)	40 (25,5)	42 (22,3)	0,11
Alcoolismo	429	26 (6,1)	9 (10,7)	11 (7,0)	6 (3,2)	0,046
Tratamentos						
Corticosteroides	429	399 (93,0)	78 (92,9)	143 (91,1)	178 (94,7)	0,426
Sedação	429	371 (86,5)	76 (90,5)	140 (89,2)	155 (82,4)	0,094
Bloqueador neuromuscular	358	294 (82,1)	54 (73,0)	109 (80,1)	131 (88,5)	0,01
Posicionamento em decúbito ventral	358	171 (47,8)	23 (31,1)	65 (47,8)	83 (56,1)	0,002
Hemodiálise	429	164 (38,2)	35 (41,7)	61 (38,9)	68 (36,2)	0,68
Óxido nítrico	358	20 (5,6)	0 (0,0)	11 (8,1)	9 (6,1)	0,048
ECMO	358	11 (3,1)	0 (0,0)	6 (4,4)	5 (3,4)	0,19
Suporte ventilatório						
CNAF	429	92 (21,5)	14 (16,7)	34 (21,8)	44 (23,4)	0,46
VNI	429	154 (35,9)	34 (40,5)	48 (30,6)	72 (38,3)	0,20
VM	429	358 (83,4)	74 (88,1)	136 (86,6)	148 (78,7)	0,06
Duração total da VM	355	15 (8 - 26)	13 (7 - 22)	15 (8 - 29)	16 (10 - 28)	0,08
Duração da VM (sobreviventes)	175	14 (7 - 27)	12 (7 - 24)	13 (6 - 30)	15 (9 - 26)	0,45
Mobilidade						
Escala de Mobilidade na alta da UTI	248	5 (4 - 7)	5 (4 - 7)	5 (4 - 7)	5 (4 - 8)	0,63
Escore Perme	236	20 ± 8	19 ± 8	20 ± 8	20 ± 8	0,73
Dias de repouso no leito	250	8 (3 - 18)	6 (2 - 16)	8 (3 - 19)	9 (3 - 20)	0,40
Mortalidade na UTI	429	182 (42,4)	52 (61,9)	57 (36,3)	73 (38,3)	0,002
Tempo de internação na UTI	429	15 (8 - 26)	13 (8 - 25)	15 (8 - 28)	14 (8 - 26)	0,43
Mortalidade hospitalar	429	195 (45,5)	57 (67,9)	61 (38,9)	77 (39,5)	< 0,001
Tempo de hospitalização	429	22 (13 - 36)	21 (13 - 34)	23 (14 - 39)	22 (13 - 35)	0,45

IMC - índice de massa corporal; SAPS 3 - *Simplified Acute Physiology Score 3*; ECMO - oxigenação por membrana extracorpórea; CNAF - cânula nasal de alto fluxo; VNI - ventilação mecânica não invasiva; VM - ventilação mecânica. Resultados apresentados como n (%), média ± desvio-padrão e mediana (IQ25 - 75%).

A taxa de mortalidade na UTI foi de 42,4% e, nos pacientes com IMC normal, foi de 61,9%. Em comparação com os pacientes com IMC normal, os pacientes com sobrepeso e obesidade tiveram maior tempo de sobrevida na UTI (*log-rank* < 0,001) (Figura 2). De acordo com o modelo de regressão multivariável de Poisson, o sobrepeso e a obesidade foram fatores de proteção significativos contra a mortalidade nas UTIs de pacientes que necessitaram de VM (p = 0,04 e p < 0,001, respectivamente). Assim, os pacientes com sobrepeso tiveram risco 36% menor de evoluir ao óbito do que os pacientes com IMC normal, enquanto nos pacientes com obesidade, a taxa foi de 23%. A pontuação SAPS 3 foi apresentada como fator de risco independente para mortalidade (p < 0,001), e cada ponto adicional na pontuação representou risco de 2% de morte na UTI para pacientes que necessitavam de VM. Na análise univariada, a idade e o diabetes foram fatores de risco para mortalidade (p < 0,001 e p = 0,02, respectivamente), mas perderam o efeito após os ajustes da análise multivariada. A duração da VM não contribuiu para a mortalidade na UTI (Tabela 2).

**Figura 2:** Curva de Kaplan-Meier do tempo de sobrevida na unidade de terapia intensiva por grupo de índice de massa corporal (*log-rank* p < 0,001). IMC - índice de massa corporal; UTI - unidade de terapia intensiva.

**Tabela 2:** Riscos relativos ajustados à associação entre a categoria do índice de massa corporal e a mortalidade somente para pacientes em ventilação mecânica

Variável	n	Mortalidade	Invariável		Multivariável	
			Risco relativo (IC95%)	Valor de p	Risco relativo (IC95%)	Valor de p
Idade	346	181	1,02 (1,01 - 1,02)	< 0,001	1,01 (1,00 - 1,02)	0,18
Feminino	346	181	0,97 (0,79 - 1,19)	0,78	0,93 (0,76 - 1,13)	0,45
Diabetes	346	181	1,26 (1,03 - 1,54)	0,02	1,19 (0,95 - 1,48)	0,13
Hipertensão	346	181	1,17 (0,94 - 1,45)	0,16	0,97 (0,76 -1,24)	0,80
SAPS 3	346	181	1,02 (1,01 - 1,03)	< 0,001	1,02 (1,01 - 1,03)	< 0,001
Duração da VM*	346	181	1,06 (0,94 - 1,20)	0,37	1,4 (1,00 - 1,29)	0,054
IMC	346	181				
18,5 - 24,99kg/m^2^			1,0 (referência)		1,0 (referência)	
25 - 29,99kg/m^^2^^			0,59 (0,46 - 0,78)	< 0,001	0,64 (0,5 0- 0,82)	< 0,001
≥ 30kg/m^^2^^			0,63 (0,49 - 0,80)	< 0,001	0,77 (0,61 - 0,97)	0,02

IC95% - intervalo de confiança de 95%; SAPS 3 - *Simplified Acute Physiology Score 3*; VM - ventilação mecânica; IMC - índice de massa corporal.

* Dias de ventilação mecânica em *log*.

No geral, 83,4% dos pacientes precisaram de VM por um tempo total de VM mediano de 15 dias (8 - 26), e não houve diferença entre os três grupos (Figura 3). Nos sobreviventes, a duração da VM não diferiu entre os grupos (Tabela 1).

**Figura 3:** Duração da ventilação mecânica em sobreviventes segundo o grupo índice de massa corporal. IMC - índice de massa corporal; VM - ventilação mecânica.

Ao comparar os dados de mobilidade, não foram observadas diferenças entre os pacientes com obesidade, sobrepeso e IMC normal (Tabela 1). A mediana de tempo para a primeira saída do leito em todos os grupos foi de 8 dias (3 - 18). No momento da alta da UTI, a pontuação média do Perme foi de 20 ± 8 pontos, indicando que uma pontuação mais alta corresponde a uma menor necessidade de assistência para mobilização. O nível de mobilidade alcançado na Escala de Mobilidade da UTI foi 5 (4 - 7), o que significa que 50% dos pacientes eram capazes de transferir ativamente o peso de uma perna para outra até uma cadeira. Não houve associação entre o IMC e a mobilidade na alta da UTI (escore Perme e Escala de Mobilidade em UTI) ou entre o IMC e o tempo de saída do leito. No entanto, o tempo de internação na UTI apresentou uma correlação inversa moderada com o escore Perme (r = -0,461, p < 0,001) (Figura 4), e o padrão dessa associação permaneceu semelhante quando os grupos foram analisados separadamente.

**Figura 4:** Correlação do Escore de Mobilidade em Unidade de Terapia Intensiva de Perme na alta da unidade de terapia intensiva com o tempo de internação na unidade de terapia intensiva (r = -0,461; p < 0,001). UTI - unidade de terapia intensiva.

## DISCUSSÃO

Este estudo unicêntrico avaliou a influência da obesidade na mortalidade, na duração da VM e no nível de mobilidade na alta da UTI em pacientes graves diagnosticados com COVID-19. Além de apresentarem taxas de mortalidade mais baixas, os pacientes com sobrepeso e obesidade também tiveram maior tempo de sobrevida na UTI do que os pacientes com IMC normal. O sobrepeso e a obesidade foram considerados fatores de proteção para prever a mortalidade na UTI após o ajuste para fatores de confusão. Não houve associação entre obesidade e duração da VM. O nível de mobilidade na alta da UTI não diferiu entre os grupos e mostrou correlação inversa moderada com o tempo de internação na UTI.

A prevalência de pacientes com obesidade internados na UTI por COVID-19 foi alta no presente estudo (43,8%), como também descrito em publicações anteriores.^(17-20)^ A obesidade é um fator independente associado a um risco aumentado de admissão à UTI por COVID-19.^(20)^ Em uma coorte retrospectiva, aproximadamente metade dos indivíduos admitidos à UTI com SARS-CoV-2 tinha IMC ≥ 30kg/m^2^, o que difere da distribuição observada em anos anteriores de hospitalização por causas pulmonares, nos quais a prevalência de obesidade foi de apenas 25,8%.^(18)^ No estudo de van der Voort et al., 90% dos pacientes infectados com SARS-CoV-2 com insuficiência respiratória tinham IMC superior a 25kg/m^2^, e a gravidade da doença aumentava significativamente com o aumento do IMC.^(21)^ Em um estudo de caso-controle 1:2 pareado, os pacientes com obesidade tinham maior probabilidade de desenvolver doença grave por SARS-CoV-2 e exigir tratamento intensivo após a exclusão de fatores de confusão.^(22)^

A obesidade pode aumentar a gravidade da doença e possivelmente predispor indivíduos mais jovens à necessidade de hospitalização e admissão à UTI.^(18,23)^ Embora não tenhamos avaliado o risco de admissão à UTI, além da alta prevalência de obesidade, os pacientes com sobrepeso eram mais jovens do que aqueles com IMC normal. Kass et al. relataram correlação inversa entre idade e IMC em uma população de 265 pacientes admitidos à UTI com COVID-19, na qual indivíduos mais jovens hospitalizados tinham maior probabilidade de serem obesos.^(24)^

A associação entre obesidade e um maior risco de morte é controversa. Alguns estudos defendem a existência de um “paradoxo da obesidade”, no qual os pacientes com obesidade têm um risco aumentado de quadro crítico, porém uma mortalidade semelhante à dos pacientes sem obesidade.^(7-10,20)^ Antes do nosso estudo, algumas coortes retrospectivas analisaram a mortalidade de pacientes obesos na UTI e, de forma semelhante aos nossos dados, constataram que a proporção de sobreviventes foi maior^(9)^ ou semelhante^(10,25-27)^ nos pacientes obesos. Essa menor taxa de mortalidade em pacientes obesos na UTI pode estar relacionada ao paradoxo da obesidade como um efeito protetor ou ao viés de seleção de pacientes com obesidade menos grave admitidos à UTI e às limitações inerentes aos estudos observacionais. As UTIs do presente estudo fazem parte do hospital de referência para assistência de alta complexidade a pacientes com COVID-19 no Rio Grande do Sul. Assim, pacientes com maior gravidade e/ou pacientes que se acredita terem maior probabilidade de complicações foram encaminhados para tratamento de alta complexidade, assim como pacientes com maior probabilidade de recuperação.

Várias metanálises examinaram a relação entre a obesidade e o aumento do risco de morte hospitalar,^(2-6,28)^ no entanto, as medidas de heterogeneidade dos resultados de morte são moderadas ou substanciais. A revisão sistemática mais recente que abordou o efeito da obesidade na mortalidade revelou evidências de alta certeza de que a obesidade de classe III está associada a um risco aumentado de morte em pacientes com COVID-19, mas em casos leves de obesidade (classes I e II), esse fator pode não estar associado de forma independente.^(28)^

Em relação à duração da VM, observamos, como em outros estudos, a necessidade de tempo prolongado em pacientes com COVID-19.^(29)^ No entanto, essa demanda por maior duração da VM não foi associada à obesidade, como no estudo de Pouwels et al. que, inclusive, relatou duração de VM semelhante aos nossos dados: 14 (8 - 23) dias.^(25)^ Kooistra et al., apesar de não terem encontrado diferença na duração da VM entre pacientes com e sem obesidade, relataram duração mediana de VM de 22 (16 - 40) dias.^(26)^

Os dados sobre mobilidade imediatamente após quadros críticos em pacientes com COVID-19 são limitados, provavelmente refletindo a natureza da pandemia, quando as prioridades são a estabilização e a sobrevida desses pacientes. O estudo de Medrinal et al. revelou que os níveis de mobilidade na alta da UTI eram semelhantes aos nossos e concluiu, usando o *Medical Research Council*, que 69% dos sobreviventes da COVID-19 na UTI desenvolveram fraqueza muscular adquirida na UTI.^(30)^ Pesquisa realizada no mesmo hospital do presente estudo revelou que pacientes críticos com COVID-19 que desenvolveram fraqueza muscular adquirida na UTI apresentaram força e mobilidade comprometidas na alta hospitalar e aumento da dependência funcional 6 meses após a alta da UTI.^(31,32)^ Estudo retrospectivo realizado na Itália destacou comprometimento funcional significativo nesse perfil de paciente, com melhora substancial na alta hospitalar, por meio de um programa de reabilitação precoce.^(33)^

Os pacientes com obesidade representam um desafio a mais para a reabilitação na UTI. O estudo prospectivo de McWilliams et al. descreveu a reabilitação de 110 sobreviventes da COVID-19 que necessitaram de VM e observou um impacto significativo do IMC no tempo até a primeira mobilização: os pacientes com IMC > 40kg/m^2^ levaram uma média de 8 dias a mais para se sentarem à beira do leito pela primeira vez, em comparação com aqueles com IMC < 25kg/m^2^.^(14)^ Esse atraso não foi observado em nosso estudo; não houve diferença no número médio de dias de repouso no leito entre os diferentes grupos de IMC, mesmo com a gravidade evidenciada pela maior prevalência de uso de bloqueadores neuromusculares e posicionamento em decúbito ventral em pacientes com IMC > 25kg/m^2^. Deve-se observar que a reabilitação começou cedo, com os pacientes ainda intubados, pois o tempo mediano de repouso no leito foi de 8 dias, e a duração mediana da VM entre os sobreviventes foi de 14 dias.

No momento da alta da UTI, 50% dos nossos pacientes atingiram o nível de mobilidade correspondente à transferência do leito para a cadeira, semelhante aos achados dos estudos de McWilliams et al. e Medrinal et al.^(14,30)^ Os níveis de mobilidade com alta dependência na alta da UTI, mesmo com o início da reabilitação precoce, podem refletir a gravidade da doença com a necessidade de VM prolongada, sedativos e bloqueadores neuromusculares. Outro fator a ser considerado é que a duração da VM e o tempo de internação na UTI é muito próximo, com medianas de 14 e 15 dias, respectivamente, o que sugere que a alta da UTI ocorre precocemente após a extubação para liberar leitos críticos devido à sobrecarga sem precedentes no sistema de saúde. A mobilidade em nosso estudo foi inversamente associada ao tempo de internação na UTI. Em estudo realizado por Timenetsky et al., os pacientes do grupo que apresentou melhor mobilidade na alta da UTI tiveram menor tempo de internação na UTI e menor duração da VM.^(34)^

Nosso estudo tem limitações. Primeiro, existe a possibilidade de confusão residual devido ao desenho observacional. A mortalidade pode ser influenciada por muitos outros fatores potencializados pela pandemia, como disparidades socioeconômicas, diretivas antecipadas e decisões de assistência médica, dificuldade de acesso ao sistema de saúde e superlotação. Em segundo lugar, não medimos a gravidade do acometimento pulmonar usando o índice de oxigenação ou outros testes. Terceiro, os dados sobre os níveis de mobilidade foram coletados somente na alta da UTI, limitando quaisquer conclusões sobre a recuperação física geral. Além disso, o estudo foi realizado em um único hospital e pode não ser representativo de outras populações. Quarto, o estudo foi realizado durante a primeira onda da pandemia, quando as vacinas contra a SARS-CoV-2 estavam em desenvolvimento. Embora tenha sido um estudo em um único centro, a instituição era o centro de referência para tratamento intensivo de pacientes com COVID-19.

Apesar dessas limitações, este estudo também tem pontos fortes notáveis: tem um tamanho de amostra considerável; usa riscos relativos ao invés de razões de chances, que são frequentemente calculadas em outros estudos e podem exagerar a razão de risco; é um dos poucos a avaliar a relação entre obesidade e mobilidade em pacientes com COVID-19; e as principais considerações de resultados referem-se exclusivamente a pacientes que concluíram sua internação na UTI, o que permite convicção nas conclusões. Pesquisas futuras devem avaliar sistematicamente a força muscular e a funcionalidade para obter melhores conclusões sobre a recuperação física geral, auxiliando ainda mais as decisões de assistência médica. Além disso, os estudos devem avaliar melhor o impacto do sobrepeso/obesidade, principalmente nos desfechos de saúde, como mortalidade, já que a maioria das evidências engloba essa categoria em indivíduos com IMC normal.

## CONCLUSÃO

Os resultados sugerem que os pacientes com sobrepeso e obesidade têm menor mortalidade e maior tempo de sobrevida na unidade de terapia intensiva do que os pacientes com índice de massa corporal normal, embora precisem mais frequentemente de terapias “agressivas” durante a infecção por SARS-CoV-2. Os pacientes com COVID-19 que necessitaram de ventilação mecânica por um período prolongado, independentemente da faixa de índice de massa corporal elevada, foram inversamente associados ao tempo de internação na unidade de terapia intensiva, mas o sobrepeso e a obesidade não influenciaram o nível de mobilidade ou assistência. Recomenda-se que os pacientes obesos sejam monitorados para evitar desfechos clínicos e funcionais desfavoráveis por meio da implementação de terapias de apoio e do início precoce de programas de reabilitação.
